# Effect of HIV status on fertility intention and contraceptive use among women in nine sub-Saharan African countries: evidence from Demographic and Health Surveys

**DOI:** 10.3402/gha.v7.25579

**Published:** 2014-10-23

**Authors:** Joyce N. Mumah, Abdhalah K. Ziraba, Estelle M. Sidze

**Affiliations:** 1 Population Dynamics and Reproductive Health Program, African Population and Health Research Centre, Nairobi, Kenya; 2 Health Challenges and Systems Program, African Population and Health Research Centre, Nairobi, Kenya

**Keywords:** fertility intention, contraception, HIV status, women, sub-Saharan Africa

## Abstract

**Background:**

Expanding access to antiretroviral therapy (ART) means that HIV is no longer a death sentence. This change has implications for reproductive decisions and behaviors of HIV-infected individuals.

**Design:**

Using multiple rounds of biomarker data from Demographic and Health Surveys (2004–2012) in nine sub-Saharan African countries, we compare patterns of associations between HIV status and fertility intention and between current use of modern contraception and HIV status in the context of expanding ART coverage.

**Results:**

Generally, results show that knowledge of HIV status and proportion of women ever tested for HIV increased substantially between the two surveys for almost all countries. Whereas modern contraceptive use slightly increased, fertility intentions remained relatively stable, except for Rwanda, where they decreased. Results from the two surveys for the nine countries do however indicate that there is no clear consistent pattern of fertility intention and modern contraceptive use behavior by HIV status, with variations observed across countries. However, multivariate analyses show that for Rwanda and Zimbabwe women who were HIV positive, with knowledge of their status, had lower odds of wanting more children. Similarly only in Rwanda (both surveys) were HIV-positive women who knew their status more likely to be current users of contraception compared with women who were HIV negative. The reverse was observed for Zimbabwe.

**Conclusions:**

Generally, the results point to the fact that the assumption that reproductive intention and behavior of HIV-positive women will differ compared with that of HIV-negative women may only hold true to the extent that women know their HIV status. Continuous expansion of voluntary counseling and testing services and integration of HIV treatment and care services with reproductive health services are thus warranted.

About 70% of the 34 million people living with HIV globally in 2011 resided in sub-Saharan Africa (SSA), with women comprising 58% of persons HIV infected (1). These disproportionately higher rates of HIV among women of reproductive ages in SSA have implications not only for health but also for life course transitions such as childbearing (2), an important marker for women in SSA.

HIV acquisition in most SSA countries is usually among young women, who are yet to start childbearing, and therefore likely to state a pregnancy desire. However, studies on fertility desires among HIV-positive women have produced varied results, indicating a complex relationship between HIV status and fertility. Numerous studies show that fear of disease progression and welfare of children influences pregnancy desires of HIV-infected women. For example, studies in Malawi, Uganda, and Zambia show that HIV status was associated with low fertility intentions and low contraceptive use (3–6). Similarly, in Malawi, desire for future children declined with HIV diagnosis (3), and the majority of newly diagnosed women in the United States chose not to become pregnant, after learning their HIV status (7). Conversely, studies in South Africa showed that potential childbearing had more of a psychological effect, as having a child was considered a reason for living, while pregnancy rates were about the same for women living with HIV and AIDS (WLHA) and the general population in Burkina Faso (8, 9).

Fears associated with childbearing in some cases can be attributed to two factors according to Laher et al. (2), the lack of educational materials on this topic, and the attitudes of health care providers toward HIV and fertility. With regard to providers, evidence from South Africa and the United States indicates that providers’ failure to relay accurate information on reproductive health to WLHA which is not tainted by their own personal bias, not only plays a major role in fear of childbearing among WLHA, but also on the decision to keep a pregnancy (7, 9). Other studies have found that factors such as income, treatment, length of clinic attendance, time since diagnosis, disclosure of status to partner and recent CD_4_ count, misinformation from peers and providers, and the potential fear of mother-to-child transmission (MTCT) reduced the desire for children among HIV-infected women (10, 11).

There is evidence indicating that increased life expectancy as a result of HAART and the associated health restoration and the reduced risk of vertical transmission to babies through prevention of MTCT, is associated with higher childbearing desires among women living in SSA. In a multicountry comparison of South Africa, Brazil, and Uganda, for instance, it was observed that sexually active women using HAART were both more likely to desire pregnancy and to practice protected sex, thereby affecting actual childbearing (12). Time since diagnosis seemed to be an especially important predictor as adjustment to recent diagnosis is yet to affect the woman's identity and wanting a child was used as a coping mechanism (11). However, the time between improved health status once on HAART may only increase fertility desire but may have no impact on actual fertility as noted by Maier et al. (13) in their study on HIV-positive women in rural Uganda.

Whether specified or not, pregnancy intentions may affect contraceptive utilization, and among women living with HIV, the decision to use contraception is complex, not unidirectional, and affected by several factors. Studies in Malawi and Uganda found, for instance, low stated desire for pregnancy among HIV-positive women, but also found low contraceptive usage (3, 4, 14). Similarly, in Zambia, there were high rates of contraceptive discontinuation among women living with HIV (15). Using nationally representative data of 10 African countries, Bankole et al. (16), however, observed high use of condoms at last sex among HIV-positive women who knew their status compared with HIV-negative women for four of the ten study countries. It is noted that among HIV-positive women, limited or non-use of contraception could be affected by health and side effect concerns, especially the fear that contraceptive use could have an impact on HIV disease progression (16). Similarly, it is argued that myths about interaction between some contraceptive methods and HAART influenced uptake of contraception (10).

In sum, there is no consensus on the associations between fertility desires, contraceptive use behavior, and HIV status. Shedding more light on these associations in SSA countries is of particular importance because HIV incidence increased by almost 31% among young women aged 15–24 (17), who are just beginning their childbearing life. Comparative studies using Demographic and Health Surveys (DHS) data (16) have only utilized one round of the data to assess these associations and it will be important to assess the changes over a more expanded period of time. This study therefore investigates the associations between HIV status and probable knowledge about HIV status on fertility intention and current contraceptive behavior for women in nine sub-Saharan African countries. We expanded the previous analyses by providing a comparison between countries and changes in these associations, using two survey periods for each country, over a span of 10 years. Findings are expected to inform service provision to meet the needs of HIV-positive women.

## Methods

### Data

This study used data collected by the DHS in nine sub-Saharan African countries that have linkable information on HIV testing, fertility preferences, and contraceptive use in at least two surveys in the same country. The countries that met the criteria are: Cameroon (2004 and 2011), Ethiopia (2005 and 2011), Guinea (2005 and 2012), Kenya (2003 and 2008/09), Lesotho (2004 and 2009), Malawi (2004 and 2010), Rwanda (2005 and 2010), Niger (2006 and 2012), and Zimbabwe (2005/06 and 2010/11).

### Design/sampling

DHS are nationally representative surveys that are carried out approximately every 5 years in several developing countries that lack data on health and social indicators. All the country surveys have similar core modules; however, other specific modules are added or modified depending on the differing needs and priorities of the countries. HIV testing has been included since the early 2000s in various countries, with most high HIV-prevalent countries having implemented at least one of the standard DHS surveys or a similar survey such as the AIDS Indicator Surveys. For this study, the standard DHS with HIV testing were used because the AIDS Indicator Surveys do not have adequate reproductive health data needed for the analysis.

The DHS uses a multistage complex cluster sampling methodology to achieve a nationally representative sample of households in the respective countries. All women of reproductive age (15–49 years) in sampled households are interviewed. Modules implemented often ask questions about household characteristics, reproductive health, contraceptive history and mortality among women of reproductive age; health, nutrition, and mortality of all children under age 5. Depending on country needs, other modules implemented include taking of biomarkers for HIV, hepatitis, syphilis and micronutrients, and sexual gender-based violence, among others. In this analysis, we used data collected in standard DHS surveys that collected blood for HIV testing and where the results were linkable to the individual woman and household level information. Data from DHS surveys and associated documentation are publicly available from the DHS measure website upon making an official application indicating intended use (www.dhsprogram.com).

### Study population

The study population consisted of women aged 15–49 who provided information on their reproductive history and blood sample for HIV testing. Normally all women in the sampled households within the age bracket and having had a birth in the last 5 years are interviewed, whereas HIV testing is done for all women in sampled households. However, because some of the eligible women did not consent to providing a blood sample for testing, the file with reproductive data did not exactly match that for HIV testing. The non-matching cases were therefore excluded from the analytical file used for analysis.

### Measures

#### Outcome variables

This study had two outcome variables: fertility intention and modern contraceptive use. Fertility intention was derived from a question to women on whether they wanted to have more children or not. For the analysis, a dichotomous variable was created with the value of 1 if a woman said ‘yes’ (i.e. she intended to have more children) and 0 if a woman said ‘no’. Women who said they were unsure were very few and dropped from the analysis. Similarly, women who indicated that they were infecund or sterilized were also excluded from the analysis. The variable for contraceptive use was derived from a question to all married or sexually active unmarried women on whether they are currently using any modern family planning method to delay or avoid getting pregnant. For the analysis, the variable takes on a value of 1 if a women said ‘yes’ and 0 if a women said ‘no’.

#### Predictor variables

The key predictor variable in this study was the woman's HIV status. HIV testing in DHS was carried out using standard protocols in accredited laboratories. Quality assurance and control was done by having all HIV-positive specimens and about 5% of the sample of HIV-negative specimens re-tested at a different laboratory using the same testing protocol (18). For the purposes of this analysis, HIV status was grouped into four categories, taking into account if the woman knew her HIV status at time of the survey. This was deemed important as it may have influenced her fertility and contraceptive use choices. The four categories were: HIV negative and knew status; HIV negative and didn't know status; HIV positive and didn't know status; and HIV positive and knew status at time of survey.

#### Control variables

In examining the associations between HIV status and probable knowledge about HIV status on fertility intention and current contraceptive behavior, our multivariate analyses included the following control variables: Current age (15–19, 20–24, 25–29, 30–34, and 35 and above), level of educational attainment (no education, primary, and secondary and above), marital status (never married, currently married, and separated/divorced/widowed), number of living children (0–1, 2–3, and 4 or more), residence (rural and urban), and household wealth status (lowest-poorest, second, middle, fourth, and highest-richest).

### Analysis

HIV sero-data were merged with each woman's individual file which also contained household variables to create an analytical file. Variables were recoded appropriately and uniformly across countries and across surveys. Country-specific analyses were carried out after merging the HIV test results and women's files, and survey-specific weights were applied in the HIV file. Survey-specific weights were used for all country surveys to account for the degree to which a woman's chances of being selected for the sample depended on household size and other DHS sampling criteria. We first present descriptive and bivariate results by country and year of survey on women's HIV status, proportion of women ever tested and tested in the survey, proportion of women who wanted more children, and those currently using contraceptive by HIV status. We also present overall HIV prevalence for each country and year of survey. We then present results from the logistic regression models on the associations between HIV status, fertility intention, and contraceptive use. All analyses were carried out using STATA 13.1, with 5% level of significance and using the ‘svy’ command to account for the complex DHS survey design.

## Results

### Descriptive and bivariate analysis

Table 1 shows the total number of women who participated in each country survey included in this study. It also presents the proportion of women who had ever tested for HIV, the proportion who were tested during the survey, their fertility preferences, and modern contraceptive use. Results show that for all countries, the proportion of women who had ever been tested for HIV substantially increased between the first and second surveys, whereas the proportion tested during the DHS remained relatively stable between surveys, except for Ethiopia and Malawi. The proportion of women who wanted to have more children in both survey years was lowest in Lesotho and highest in Niger. Generally, current modern contraceptive use was higher in the second survey in all countries, with the highest levels observed in Zimbabwe and lowest observed in Guinea.

**Table 1:** Percentage distribution of women ever tested for HIV, tested during survey, want more children and modern contraceptive use in the two surveys, per study country

Country	Survey year	Proportion ever tested	Proportion tested	Want more children	Modern contraceptive use	Number of women in sample
Cameroon	2004	20.7	47.9	82.2	14.0	10,656
	2011	24.6	46.8	78.2	16.2	15,426
Ethiopia	2005	1.6	43.4	60.4	9.7	14,070
	2011	35.8	95.2	68.0	18.7	16,515
Guinea	2005	2.1	48.4	79.6	6.8	7,954
	2012	10.5	51.6	84.1	7.0	9,142
Kenya	2003	13.1	40.7	61.8	22.7	8,195
	2008/09	56.5	45.4	59.3	28.0	8,444
Lesotho	2004	12.0	41.8	47.1	27.6	7,095
	2009	65.6	49.7	44.6	34.9	7,624
Malawi	2004	12.9	23.4	60.8	22.4	11,698
	2010	71.6	31.8	63.0	32.6	23,020
Niger	2006	1.9	48.7	91.0	4.5	9,223
	2012	21.3	46.0	91.8	11.0	11,160
Rwanda	2005	21.4	50.2	65.8	5.7	11,321
	2010	75.5	50.8	47.5	25.2	13,671
Zimbabwe	2005/06	21.7	84.0	61.6	39.1	8,907
	2010/11	57.4	86.1	63.3	40.5	9,171

Table 2 shows the percentage distribution of HIV status taking into account prior knowledge of HIV status from two consecutive surveys. Table 2 also presents results for the overall HIV prevalence and overall prior knowledge of HIV status from consecutive surveys for each country. In the first survey for each country, the majority of women were HIV negative and did not know their status. However, in the subsequent survey, this proportion greatly reduced for all countries, especially in Lesotho, Malawi, Rwanda, and Zimbabwe. For all countries, the proportion of women who were HIV positive and knew their status increased between surveys, particularly in Lesotho, Zimbabwe, and Malawi. Overall, among women who were tested for HIV in each survey, there was a substantial increase in the proportion of women who knew their status, irrespective of whether the results were negative or positive. The overall HIV prevalence for all countries did not change substantially between the inter-survey years, except for Zimbabwe where a slight decrease was observed.

**Table 2:** Overall HIV prevalence, HIV status and prior knowledge of status among women tested for HIV in the two surveys, per study country

Country	Survey year	HIV −ve; knew status	HIV −ve; didn't know status	HIV +ve; didn't know status	HIV +ve; knew status	Overall HIV prevalence	Prior knowledge of HIV status	Number
Cameroon	2004	17.9	75.5	4.7	1.9	6.6	19.8	5,154
	2011	47.2	47.2	1.7	3.9	5.6	51.1	7,253
Ethiopia	2005	3.2	95.0	1.7	0.2	1.9	3.4	5,942
	2011	34.7	63.5	0.5	1.3	1.9	36.0	15,505
Guinea	2005	1.6	96.5	1.8	0.1	1.9	1.7	3,842
	2012	9.8	88.1	1.8	0.3	2.1	10.1	4,689
Kenya	2003	11.7	79.6	7.1	1.6	8.7	13.3	3,271
	2008/09	52.0	40.1	2.1	5.9	8.0	57.8	3,811
Lesotho	2004	7.2	66.4	22.0	4.4	26.4	11.7	3,020
	2009	45.9	27.4	7.8	18.9	26.7	64.8	3,849
Malawi	2004	9.9	76.8	11.5	1.8	13.3	11.7	2,864
	2010	61.5	25.7	2.5	10.4	12.9	71.9	7,396
Niger	2006	1.8	97.5	0.7	0.0	0.7	1.8	4,441
	2012	19.9	79.7	0.2	0.2	0.4	20.1	5,101
Rwanda	2005	19.4	77.0	2.1	1.5	3.6	20.8	5,663
	2010	71.6	24.7	0.3	3.4	3.7	75.0	6,952
Zimbabwe	2005/06	16.6	62.3	15.6	5.6	21.1	22.1	7,494
	2010/11	46.0	36.3	5.1	12.6	17.7	58.6	7,852

Table 3 shows the proportion of women who wanted more children by HIV testing status among women tested and those not tested for HIV for each country. The percentages are weighted factoring the survey design in each of the surveys.

**Table 3:** Proportion of women who wanted more children by HIV testing status and prior knowledge of HIV status among women tested and those not tested for HIV in the two surveys, per study country

		Tested for HIV in survey						Not tested for HIV in survey	
				
Country	Survey year	HIV −ve; knew status	HIV −ve; didn't know status	HIV +ve; didn't know status	HIV +ve; knew status	Total	Number	%	Number
Cameroon	2004	77.9	83.4	80.8	80.6	82.2**	5,154	82.2	5,502
	2011	77.4	81.9	77.4	70.9	79.3***	7,253	77.3	8,173
Ethiopia	2005	81.0	62.0	59.2	54.6	62.6***	5,942	58.8	8,128
	2011	70.4	67.0	59.5	57.3	68.0*	15,505	70.6	1,010
Guinea	2005	88.2	80.1	69.7	76.5	80.1	3,842	79.1	4,112
	2012	83.8	83.9	79.0	96.7	83.9	4,689	84.3	4,453
Kenya	2003	58.4	63.3	53.7	61.1	62.0*	3,271	61.8	4,924
	2008/09	54.0	65.6	42.2	43.0	57.7***	3,811	60.6	4,633
Lesotho	2004	44.8	48.6	41.4	38.3	46.0*	3,020	47.9	4,075
	2009	45.0	54.8	36.9	34.1	45.0***	3,849	44.4	3,775
Malawi	2004	65.9	61.1	52.4	47.7	60.2*	2,864	60.9	8,834
	2010	65.2	74.0	49.4	39.9	64.4***	7,396	62.3	15,624
Niger	2006	88.6	90.6	89.8	100.0^a^	90.6^a^	4,441	91.3	4,782
	2012	92.8	91.3	100.0^a^	87.9	91.6^a^	5,101	92.0	6,059
Rwanda	2005	65.2	67.5	46.0	35.9	66.1***	5,663	65.5	5,658
	2010	50.1	44.8	48.4	30.3	48.6***	6,952	46.4	6,719
Zimbabwe	2005/06	61.9	68.0	41.1	40.7	61.3***	7,494	62.9	1,413
	2010/11	62.6	74.2	49.7	33.7	62.5***	7,852	66.3	1,319

Chi-squared statistic assesses the significant differences between the different knowledge status categories and fertility preferences.

* *p*<0.05

** *p*<0.01,

*** *p*<0.001.

a Small numbers <*5 HIV positive cases* in the cells.

With the exception of Guinea and Niger, the percentage distribution of women who wanted more children significantly varied by one's HIV status and knowledge of the status (*p* < 0.05). This was more so the case with the second survey in each of the countries. For example in the 2003 Kenya survey, 61% of women who were HIV positive and knew their status prior to the survey wanted to have more children. In the 2008/09 Kenya survey, the proportion of women who were HIV positive and knew their status prior to the survey and wanted more children dropped to 43%. Similar trends were observed in Malawi and Zimbabwe where the proportion of women who were HIV positive and knew their status and wanted more children was 48% in 2004 and 41% in 2005/06, but declined to 40% in 2010 and 34% in 2010/11, respectively. Results also show that the proportion of women who wanted more children among those tested for HIV were generally similar to those of women not tested for HIV for all surveys. However, fertility preferences seemed to be predicated by a combination of testing and knowledge of status in some countries, as HIV-positive women with knowledge of their status for both surveys in Lesotho, Malawi, Rwanda, and Zimbabwe were less likely to want more children, compared with women not tested for HIV.

Table 4 shows the proportion of women currently using a modern contraceptive method by HIV testing status among women tested and those not tested for HIV for each study country. Generally, with exception of Guinea, current contraceptive use in the countries studied was higher in their later survey than the earlier survey. The percentage distribution of current contraceptive use among women significantly varied by HIV status and knowledge of status in each of the surveys in the countries studied. The patterns varied but it can be observed that for Cameroon (2004), Guinea (2005), and Malawi (2004), the proportions of women who were HIV negative and knew their status and used a modern contraceptive were higher compared with women who were HIV positive and knew their status. Similar patterns were observed in the most recent surveys in five countries (Cameroon, Kenya, Malawi, Niger, and Zimbabwe). On the other hand, Ethiopia, Guinea and Lesotho (second surveys), Niger (2006), and Rwanda (2005), the proportion of women using a modern contraceptive was higher among women who were HIV positive and knew their status. Additionally, estimates show that the proportion of women currently using a modern contraceptive method among those tested for HIV was generally similar to levels among women not tested for HIV in all surveys. Current contraceptive use also seemed to be predicated by a combination of testing and knowledge of status, in that, compared with women not tested for HIV in the respective surveys, HIV-positive women with knowledge of their HIV status were more likely to report being current users of a modern contraceptive method in all study countries.

**Table 4:** Proportion of women who were using modern contraception by HIV testing status and prior knowledge of HIV status among women tested and those not tested for HIV in the two surveys, per study country

			Tested for HIV in survey					Not tested for HIV in survey	
					
Country	Survey year	HIV −ve; knew status	HIV −ve; didn't know status	HIV +ve; didn't know status	HIV +ve; knew status	Total	Number	%	Number
Cameroon	2004	23.6	12.0	14.2	21.2	14.4***	5,154	13.8	5,502
	2011	22.4	9.2	10.3	15.9	15.7***	7,253	16.6	8,173
Ethiopia	2005	18.4	9.6	26.7	61.9	10.2***	5,942	9.4	8,128
	2011	26.9	14.4	22.8	29.0	19.0***	15,505	15.3	1,010
Guinea	2005	13.9	6.8	16.7	11.4	7.1**	3,842	6.7	4,112
	2012	13.4	5.9	10.0	37.3	6.8***	4,689	7.3	4,453
Kenya	2003	35.2	20.7	23.6	35.8	22.9***	3,271	23.0	4,924
	2008/09	36.0	18.1	27.8	24.9	28.0***	3,811	27.9	4,633
Lesotho	2004	40.3	22.9	34.1	38.1	27.4***	3,020	28.2	4,075
	2009	38.8	18.4	38.6	43.5	34.1***	3,849	35.8	3,775
Malawi	2004	30.0	23.1	19.1	18.2	23.2*	2,864	22.3	8,834
	2010	38.6	19.8	18.3	36.1	33.0***	7,396	32.5	15,624
Niger	2006	13.6	4.8	6.3	21.7	5.0**	4,441	4.2	4,782
	2012	24.1	8.1	8.6	16.0	11.3***	5,101	10.9	6,059
Rwanda	2005	10.7	4.1	6.8	16.7	5.7***	5,663	5.7	5,658
	2010	31.7	5.3	15.4	37.1	25.3***	6,952	25.2	6,719
Zimbabwe	2005/06	52.7	35.8	39.4	42.4	39.5***	7,494	37.0	1,413
	2010/11	53.4	24.9	35.6	47.1	41.3***	7,852	36.6	1,319

Chi-squared statistic assesses the significant differences between the different knowledge status categories and modern contraceptive use.

* *p*<0.05

** *p*<0.01

*** *p*<0.001.

### Multivariate analysis

Table 5 shows results from logistic regression. The estimates presented for the effects of HIV status on fertility intention and current contraceptive use are net of the effects of age, wealth status, residence, education, marital status, and number of living children.

**Table 5:** Results from logistic regression models on the effect of HIV status on fertility preference and modern contraceptive use, net of other factors

		Wanted more children			Using a modern contraceptive method		
			
Country	Survey year	HIV −ve; didn't know status	HIV +ve; didn't know status	HIV +ve; knew status	HIV −ve; didn't know status	HIV +ve; didn't know status	HIV +ve; knew status
Cameroon	2004	1.13 (0.86, 1.49)	1.06 (0.64, 1.74)	0.90 (0.49, 1.66)	0.90 (0.74, 1.10)	0.77 (0.49, 1.19)	0.93 (0.53, 1.64)
	2011	1.03 (0.85, 1.26)	1.13 (0.62, 2.08)	0.98 (0.66, 1.46)	0.62*** (0.51, 0.75)	0.60 (0.31, 1.17)	0.67* (0.46, 0.97)
Ethiopia	2005	0.58* (0.35, 0.93)	0.81 (0.31, 2.11)	0.39 (0.12, 1.27)	0.75 (0.41, 1.37)	1.28 (0.50, 3.29)	13.62* (1.70, 108.85)
	2011	1.15 (0.98, 1.35)	1.31 (0.68, 2.50)	0.95 (0.43, 2.07)	0.59*** (0.50, 0.69)	0.76 (0.35, 1.65)	0.72 (0.33, 1.54)
Guinea	2005	0.66 (0.25, 1.74)	0.34 (0.11, 1.07)	0.29 (0.03, 2.80)	1.06 (0.51, 2.20)	1.87 (0.70, 5.01)	0.88 (0.10, 8.07)
	2012	1.11 (0.72, 1.70)	0.83 (0.40, 1.70)	3.38 (0.56, 20.56)	0.78 (0.56, 1.10)	1.06 (0.37, 2.99)	3.22* (1.20, 8.66)
Kenya	2003	1.37* (1.00, 1.86)	1.24 (0.75, 2.06)	1.54 (0.54, 4.41)	0.72* (0.55, 0.95)	0.60* (0.38, 0.96)	0.95 (0.44, 2.07)
	2008/09	1.47* (1.06, 2.04)	1.18 (0.48, 2.93)	0.78 (0.44, 1.39)	0.73* (0.55, 0.96)	0.81 (0.45, 1.46)	0.54* (0.32, 0.90)
Lesotho	2004	1.26 (0.83, 1.92)	1.14 (0.72, 1.80)	0.89 (0.45, 1.78)	0.61* (0.41, 0.91)	0.77 (0.50, 1.21)	0.76 (0.40, 1.47)
	2009	1.52*** (1.20, 1.92)	1.07 (0.77, 1.49)	0.96 (0.71, 1.29)	0.59*** (0.47, 0.74)	0.93 (0.66, 1.31)	1.04 (0.85, 1.27)
Malawi	2004	1.07 (0.71, 1.61)	0.65 (0.41, 1.04)	0.38 (0.14, 1.05)	0.80 (0.53, 1.21)	0.60 (0.36, 1.01)	0.48 (0.21, 1.10)
	2010	0.92 (0.74, 1.15)	0.65 (0.40, 1.04)	0.41*** (0.32, 0.53)	0.74*** (0.62, 0.88)	0.41*** (0.25, 0.65)	0.89 (0.69, 1.13)
Niger	2006	0.69 (0.23, 2.03)	1.23 (0.19, 7.75)	–	1.07 (0.58, 1.99)	0.90 (0.14, 5.88)	3.11 (0.27, 35.65)
	2012	0.65** (0.47, 0.89)	–	0.57 (0.06, 5.21)	0.43*** (0.34, 0.54)	0.38 (0.05, 2.83)	0.77 (0.10, 5.63)
Rwanda	2005	1.08 (0.88, 1.33)	0.79 (0.49, 1.28)	0.49* (0.28, 0.85)	0.73* (0.54, 0.98)	1.08 (0.43, 2.72)	2.36** (1.29, 4.32)
	2010	1.09 (0.79, 1.51)	2.23 (0.66, 7.60)	0.22*** (0.14, 0.34)	0.42*** (0.32, 0.55)	0.88 (0.24, 3.24)	1.69** (1.22, 2.33)
Zimbabwe	2005/06	1.33** (1.10, 1.62)	0.64*** (0.50, 0.82)	0.57*** (0.41, 0.78)	0.80* (0.67, 0.96)	0.65*** (0.51, 0.83)	0.61** (0.42, 0.88)
	2010/11	1.49*** (1.26, 1.77)	0.96 (0.68, 1.35)	0.36*** (0.29, 0.44)	0.55*** (0.48, 0.64)	0.63*** (0.50, 0.81)	0.98 (0.81, 1.17)

Reference category is HIV negative and knew status. Models controlled for age, wealth status, residence, education, marital status and number of living children.

* *p*<0.05

** *p*<0.01

*** *p*<0.001.

Although not statistically significant for all countries, generally, women who were HIV positive and knew their status had lower odds of wanting more children compared with HIV-negative women who knew their status before the survey, with exception of Guinea (2012) and Kenya (2003). We do, however, interpret this result with some caution because of the wide confidence intervals observed. The lower odds were particularly significant for both surveys in Rwanda and Zimbabwe and the earlier survey in Malawi. Estimates also show that women who were HIV positive and didn't know their status were not significantly different from those who were HIV negative and knew their status in terms of wanting more children with the exception of Zimbabwe (2005/06) where this was 36% lower. In Kenya (2003 and 2008/09), Lesotho (2009), and Zimbabwe (2005/06 and 2010/11), HIV-negative women who did not know their status had higher odds of wanting more children compared with women who were HIV negative and knew their status. Conversely, HIV-negative women who didn't know their status in Ethiopia (2005) and Niger (2012) had lower odds of wanting more children compared with women who were HIV negative and knew their status in these two countries.

With the exception of Guinea, estimates show that women who were HIV negative and didn't know their status were less likely to be using a modern contraceptive method compared with those who were HIV negative and knew their HIV status. The estimates were statistically significant for the two surveys for Zimbabwe, Rwanda, Lesotho, and Kenya. For Zimbabwe, Malawi (latest survey), and Kenya (earlier survey), women who were HIV positive and didn't know their status had lower odds of using a modern contraceptive method compared with those who were HIV negative and knew their status. In both surveys in Rwanda, Ethiopia (earlier survey), and Guinea (latest survey), women who were HIV positive and knew their status were more likely to report use of a modern contraceptive method compared with HIV-negative women who knew their status. The reverse was however true for the earlier survey in Zimbabwe and latest surveys in Kenya and Cameroon.

## Discussion

This study uses two rounds of DHS data from nine sub-Saharan African countries to examine the associations between HIV status, women's fertility intention, and current use of modern contraception. We particularly explored if women's desire for more children and contraceptive use by HIV status has changed in the last 10 years, in the context of expanding coverage of antiretroviral therapy (ART). Results from the two surveys for the nine countries indicate that there is no clear consistent pattern of fertility intention and modern contraceptive use behavior by HIV status after controlling for several confounding factors, with variations observed across countries.

Generally, few similarities can be noted in some of the countries. Except for women in Kenya and Guinea, women who were HIV positive and had prior knowledge of their status reported lower desires for more children than women who were HIV negative and had prior knowledge of their status. This pattern was particularly consistent for HIV-positive women who also know their status in countries such as Rwanda, Zimbabwe, and Malawi who prefer not to have more children compared with women who were HIV negative and knew their status. The three countries, in common, have relatively high HIV prevalence rates among women age 15–49 years, which ranged from 4% in Rwanda to 13% and 18% in Malawi and Zimbabwe, respectively (19–21). On the other hand, countries like Kenya, Lesotho, and Cameroon with similar HIV infection burden showed no difference in fertility intention by HIV status.

With respect to modern contraceptive use, patterns also remained inconsistent. Generally, in the recent surveys, women who were HIV negative and did not have prior knowledge of their status were less likely to be using a modern contraceptive method than those who were HIV negative and knew their HIV status. For three of the study countries (Zimbabwe, Kenya, and Malawi), net the effect of control variables, HIV-positive women with prior knowledge of their status were less likely to be current modern contraceptive users compared with women who were HIV negative and had prior knowledge of their status. Our bivariate analysis also shows that compared with women in the DHS sample for all countries who were not tested for HIV, HIV-positive women who had prior knowledge of their status were more likely to report current modern contraceptive use. For HIV-negative women without prior knowledge of their status, it is possible that the low perceived exposure to risk of contracting HIV and ambivalence toward childbearing may explain the lower odds of using modern contraceptive methods, including male condoms (16). Bankole et al. (16), in their study using one round of DHS surveys in 10 sub-Saharan African countries, argue that it is the combination of one's status and knowledge of that status that strongly influences reproductive intentions and behavior of HIV-positive individuals. Although HIV testing has expanded considerably in many sub-Saharan African countries, especially among women (confirmed by our study results), levels of HIV testing still remain relatively low in many countries (22). The expectation, therefore, that the reproductive intention and behavior of HIV-positive women will differ compared with that of HIV-negative women may only hold true to the extent that women know their HIV status.

Few countries provided a clear and consistent pattern with regards to fertility intention. Rwanda is one of the countries with a consistent pattern. Specifically, Rwandan women who were HIV positive and knew their status were less likely to want more children and more likely to be using a modern contraceptive method. The reverse is observed in Zimbabwe: women who were HIV positive and knew their status preferred not to have more children, but were also less likely to be using a modern contraceptive method compared with those who were HIV negative and knew their status. Homsy et al. (4), in their study in Uganda, suggest that the disconnect between low fertility desire and low contraceptive uptake could probably be that the responses to questions on fertility desires could be influenced by social desirability or stigma surrounding childbearing for HIV-positive individuals. Other authors have argued that low contraceptive use among HIV-positive women was based on the perception that they and their partners were infertile due to HIV infection (23).

In the case of Rwanda, by 2010, when they collected their most recent DHS survey, it was estimated that its ART coverage had reached about 80% of adults eligible for ART (24). In contrast, for countries like Malawi and Zimbabwe, estimates by 2010 show that 9 out of 10 people have an unmet need for ART (24). In fact, for many of the countries in this study, the unmet need for ART is quite high. Although speculative because of the cross-sectional nature of the data, it is possible that the observed non-consistent pattern of associations between women's positive HIV status (and knowledge of the status) and reproductive desire and use of contraception is a reflection of the differential levels of ART access and regional coverage between and within countries. This could also be a reflection of attitudes and behaviors during a pre-ART period (25).

There is no consensus on the impact of ART on fertility desires, intention, and behavior among HIV-positive individuals. Some studies show that significant proportions of HIV-positive individuals who are on ART prefer having children (3, 11, 13, 26); whereas others show the opposite effect (2). For example, studies in Uganda and South Africa show that fertility desires and sexual activity increased among HIV-positive individuals after ART initiation, though these desires were more likely to be greater among men (4, 26). On the other hand, smaller studies using longitudinal data show that HIV status was a significant predictor of fertility behavior among HIV-positive women who were more likely to change their fertility intention from wanting more children to wanting no more children (14). However, these studies also show that ART may increase desire for children but the odds of actual childbearing did not change considerably (13), suggesting that fertility intention and actual childbearing may not be correlated. Kongnyuy and Wisysonge (27), in their study on Cameroon, observed that actual fertility rates were lower among HIV-positive women compared with HIV-negative women, although fertility desires were comparable. However, considering that ART coverage is just now solidly expanding in much of sub-Saharan Africa, it is possible that the impact of ART on fertility patterns and behavior may actually change in many of the sub-Saharan Africa countries (13). In fact, several studies have documented that ART users are more likely to be using contraception compared with non-ART users (12, 28). The extent to which long-term ART access will impact reproductive decisions and behavior among HIV-positive individuals will need to be captured through long-term cohort studies. Kaida et al. (29) argue that access to HIV treatment could serve as a crucial entry point as regular access could positively influence reproductive behaviors of HIV-positive individuals. It is therefore possible that the current data is capturing a phase of early access to ART and the significant and sustained effect of ART on fertility desires, intentions, and associated behaviors will only be felt with longer term expanded coverage.

Generally, findings from this study provide support for the need for continuous expansion of voluntary counseling and testing services and integration of HIV treatment with sexual and reproductive health services (6, 14, 30). A cohort study among HIV-positive Malawian women showed, for instance, that the combination of a notification of an HIV-positive status and family planning counseling decrease future fertility intentions and increased uptake of a contraceptive method (3). Similarly, HIV-positive women with no desire to have children were also less likely to have become pregnant during the 1-year study period (3). Integrating family planning, maternal and child health, and HIV care and treatment services will therefore provide opportunities to not only influence reproductive decision-making and behaviors, but also address the fluid nature of individuals’ fertility desires especially within the context of an HIV diagnosis. Similarly, integration of HIV care and treatment services with family planning services will allow for the continuous follow-up of HIV-positive women so there is a sustained, rather than intermittent impact on their behaviors (29).

There might also be a need to address provider attitudes and biases toward the provision of services to HIV-positive individuals. The inconsistent pattern between fertility desires and contraceptive behavior could be reflective of perceived disapproval by HIV-positive individuals of society's attitude toward their fertility decisions and reproductive behavior, especially childbearing (9–11, 31, 32). There is the need to improve dialogue between providers and clients, as well as provision of accurate information, range of methods, and information on the potential side effects of these methods (2, 10, 33).

This study has two main limitations to keep in mind while interpreting the results. First, the cross-sectional nature of the data limits our ability to determine causality. As noted above, longitudinal data would allow for clearer elucidation of the mechanisms through which HIV-positive individuals make key reproductive decisions. Second, although we control for marital status, lack of data on male partners limited our ability to assess the impact of HIV status on women's fertility intention while controlling for their partners’ fertility intention. As noted in several studies, there is the need to assess the role of couple's decision-making in reproductive behavior as it has been shown that partner's attitude and fertility intentions significantly influences the woman's fertility intentions (14, 34).

## Conclusions

Despite these limitations, the study results have important implications for research. The findings add to the literature on the fact that there is no clear observed pattern between fertility intentions and contraceptive behavior among HIV-positive women in the last decade in sub-Saharan African countries. There is thus a need for continuous effort and advocacy on reaching HIV-positive women with crucial information and services that would allow them to make informed choices about the kind of reproductive lives they want to live. What remains unclear from the findings in this study and many extant studies are the clear mechanisms through which HIV-positive individuals make decisions about their reproductive life. Considering that reproductive decisions by HIV-positive individuals do not happen in a vacuum and are influenced by several factors and at various levels (individual, family, community, society, etc.), additional research is needed to clearly elucidate the pathways through which these decisions are made. This will then allow for more targeted and effective interventions that will have more sustained impact on the reproductive lives of HIV-positive individuals.
